# Microarray data mining: A novel optimization-based approach to uncover biologically coherent structures

**DOI:** 10.1186/1471-2105-9-268

**Published:** 2008-06-06

**Authors:** Meng P Tan, Erin N Smith, James R Broach, Christodoulos A Floudas

**Affiliations:** 1 Department of Chemical Engineering, Princeton University, NJ, USA; 2 Molecular and Cellular Biology Program, University of Washington, WA, USA; 3 Department of Molecular Biology, Princeton University, NJ, USA

## Abstract

**Background:**

DNA microarray technology allows for the measurement of genome-wide expression patterns. Within the resultant mass of data lies the problem of analyzing and presenting information on this genomic scale, and a first step towards the rapid and comprehensive interpretation of this data is gene clustering with respect to the expression patterns. Classifying genes into clusters can lead to interesting biological insights. In this study, we describe an iterative clustering approach to uncover biologically coherent structures from DNA microarray data based on a novel clustering algorithm EP_GOS_Clust.

**Results:**

We apply our proposed iterative algorithm to three sets of experimental DNA microarray data from experiments with the yeast *Saccharomyces cerevisiae *and show that the proposed iterative approach improves biological coherence. Comparison with other clustering techniques suggests that our iterative algorithm provides superior performance with regard to biological coherence. An important consequence of our approach is that an increasing proportion of genes find membership in clusters of high biological coherence and that the average cluster specificity improves.

**Conclusion:**

The results from these clustering experiments provide a robust basis for extracting motifs and trans-acting factors that determine particular patterns of expression. In addition, the biological coherence of the clusters is iteratively assessed independently of the clustering. Thus, this method will not be severely impacted by functional annotations that are missing, inaccurate, or sparse.

## Background

DNA microarray technology allows investigators to monitor simultaneously the expression behavior of essentially all the genes within an entire genome and can provide information on gene functions and transcriptional networks. However, the large number of genes and the complexity of the underlying biological networks make extracting this information a formidable task. A common first step to interpret DNA microarray data is to cluster the data on the basis of similarity of expression patterns. Since genes with similar function often show a common expression pattern, clustering genes of known functions with poorly characterized genes provides a means of gaining insights into the functions of the latter [1]. Furthermore, patterns seen in genome-wide expression data can reveal potential gene regulation networks and relate cellular processes to changes in cellular conditions [2,3]. However, the extent to which clustering reveals useful information about the system under study depends on the extent to which the clustering method successfully groups intrinsically related elements. Example classes of clustering algorithms include (a) single and complete link hierachical clustering [4], (b) K-family of clustering algorithms [5-7], (c) optimization-based clustering approaches [8-10], (d) fuzzy clustering [11,12], (e) quality threshold clustering (QTClust) [13], (f) artificial neural networks for clustering, such as the self-organizing map (SOM) [14] and a variant that combines the SOM with hierachical clustering, the self-organizing tree algorithm (SOTA) [15], (g) information-based clustering [16,17], and (h) stochastic approaches such as clustering by simulated annealing [18,19]. Some of these algorithms, while novel in their own rights, suffer from certain shortcomings. For instance, the K-clustering methods require a user-specified cluster number. The QTClust approach is computationally expensive and assumes at each iteration that the largest cluster formed is necessarily the best grouping. And the simulated annealing approach requires a consistently good cooling schedule, subjective inputs for the upper bound cutoff distance and the allowable lower bound false negatives, and can become computationally more expensive than exhaustive search algorithms. Whichever the clustering algorithm used, we need an intuitive and relevant tool to first assess the quality and significance of the clusters formed.

One approach to assess cluster quality is based on the idea that if the clustering result reflects robust structures, existing clusters should accurately estimate the appropriate cluster labels for new data points [20]. [21] extend the prediction strength concept by proposing a figure of merit (FOM) measure, which describes the mean deviation of the gene expression levels with respect to the pertinent cluster centers. Other methods of measuring cluster validity have also been proposed, such as the Davies-Bouldin Validity Index [22], which is a function of the ratio of the sum of within-cluster scatter to between-cluster separation.

These approaches all rely solely on mathematical coherence to assess cluster quality. However, in uncovering biologically coherent structures from DNA microarray data, categorizing gene clusters on the basis of known functionally related groups is very relevant. This provides useful insights into key biological themes among the genes and enables progress in the studies of gene regulatory networks and signal transduction pathways [23]. The biological coherence of each cluster can be scored according to the percentage of its genes covered by annotations significantly enriched in the cluster in question, using functional classification schemes in Martinsried Institute of Protein Sciences (MIPS) or the Gene Ontology (GO) databases [24,25] to generate a p-value reflecting the likelihood that such enrichment would happen by chance. Auxiliary to this central issue is the question of rigorously identifying and isolating outlier genes since it is highly probable that only a subset of genes participate in any cellular process or biological studies of interest. On the other hand, genes with similar expression profiles may not have common functional characteristics or expression profiles of genes in the same functional category may still be dissimilar due to the existence of unknown functional sub-categories and sparse or inaccurate functional annotations.

A remedy then is to integrate known biological knowledge into the clustering procedure itself. Most knowledge-based clustering methods directly incorporate GO knowledge into the algorithm. This assumes that the current GO knowledge is correct because genes known to have a similar functional annotation are 'pushed' more closely to one another in a biased fashion. However, this could handicap the clustering process if the organism is sparsely annotated. Methods that work solely on a modified distance measure [26] can thus over-estimate the distances of the genes with unknown functions, thus depriving them of a chance of being 'discovered'. An alternative is to modify the similarity measure to be a linear combination of the expression profile similarity and functional similarity. This would however not work well with organisms that are not well annotated and is always highly subjective as to the contribution score of each similarity measure. Another approach allows genes sharing common functions to have a common prior probability as compared to genes with different functions [27]. In an earlier work [28], the clustering process is regulated by must-link constraints, which apply to genes with known common functions, and cannot-link constraints, which apply to genes known not to be associated with one another. This class of knowledge-based clustering algorithms could distort the 'chance' of genes with unknown functions to be fairly clustered. It also assumes that the current GO annotations are accurate and comprehensive enough. A recent novel approach for knowledge-based clustering [29] involves the use of selectively snipping the edges of a typical hierarchical clustering tree to induce clusters that are maximally consistent with available background information such as functional annotations. This method is tested and reported to outperform another recent knowledge-based clustering method [30].

In addressing the various aforementioned concerns of many current knowledge-based clustering methods, we choose not to interfere with the measured distances of the genes or data points and instead cluster them based on the fundamental intuition that genes in the same cluster should behave similarly. Then, we introduce a secondary refinement process using the GO annotations to check the level of biological coherence of the clusters. We use the entire set of yeast GO annotations available throughout our study. The iterative nature of our procedure means that if the GO annotations are somehow wrong to begin with, subsequent iterations of our algorithm will still show a strong persistence in pushing seemingly 'unrelated' genes together, thus giving a hint that maybe these genes should have a common function after all. If the GO annotations are unknown, then the unknown genes would be still fairly clustered with their counterparts, as their measured experimental behavior has not been tampered with. In organisms that are sparsely annotated, the emphasis on these intuitive factors such as unaltered distances and response correlation provides opportunities for (a) genes with known functions and verified with similar experimental behavior to be clustered together and (b) genes with unknown functions to find cluster membership in clusters with known functions if they correlate well or in clusters with unknown functions containing counterparts that show similar expression behavior.

Our iterative clustering approach has as its backbone an optimization-based clustering algorithm, EP_GOS_Clust, presented in an earlier paper [10]. The algorithm is based on a variant of the GBD algorithm, the Global Optimum Search (GOS) [31-33]. This is a robust algorithm that compares favorably with many commonly-used clustering algorithm in defining clusters that are as dissimilar from one another as possible (that is, a large inter-cluster error sum) while assigning members that are as similar to one another as possible into the same cluster (that is, a small intra-cluster error sum). It also incorporates a methodology to predict the optimal number of clusters for a given dataset. In measuring the biological coherence of the clusters, we assert that the appropriate performance indicators are the cluster p-values and the proportion of genes that are in clusters of high coherence quality. The first reflects the functional richness of a cluster and is an intuitive measure of coherence, while the second reflects the potential to extract the maximum amount of useful information for subsequent studies of motif analysis and regulatory structure searches. We also look at cluster correlation and functional specificity as consistency checks. We then test our proposed method on three datasets of actual DNA microarray expression results.

## Results and discussion

### Outline of proposed approach for uncovering biological coherence

Our iterative approach extends a recently-proposed algorithm, the EP_GOS_Clust, as a backbone for clustering the expression data. Specifically, we perform an initial clustering run on a given data as previously described to reach the optimal number of clusters [10] and then apply a GO analysis of the data to obtain a preliminary assessment of the level of biological coherence. Those clusters that exhibit better biological coherence than a prescribed benchmark value are retained as seeds for the next round of clustering. As a consistency check, we also look at cluster correlation and specificity. Those genes that fall into clusters lacking the benchmark level of coherence are subjected to a subsequent round of EP_GOS_Clust clustering, in which each gene is placed either in an existing cluster having a similar expression profile or aggregated with other unclustered genes having similar expression profiles to form a new cluster. This overall process is repeated until we observe an asymptotic saturation either in the optimal number of clusters or the proportion of genes that are placed into clusters with strong levels of biological coherence. The approach is described in greater detail in the Methods section.

### Dataset I

Dataset I consists of DNA microarray data obtained from a study in the role of the Ras/protein kinase A pathway (PKA) on glucose signaling in yeast [34]. The Ras/PKA signal transduction pathways provide a major conduit to couple cellular responses to the availability of carbon sources such as glucose [35-37]. [34] measure the levels of RNA for each of the 6237 yeast genes over time in wild type and various mutant strains following glucose addition to cells grown on a non-fermentable carbon source. These experiments are designed to assess the extent to which the Ras/PKA pathway mediated transcriptional effects induced by glucose. [See additional file 5]

Each of the eight test and control experiments consist of four time points over a hour period, yielding 32 data points for each of the 6237 genes. Before clustering the array data, we filter the data to remove unreliable data. In particular, we retained all genes for which all the time points are deemed present by the Affymetrix software suite (4105 genes), all the genes for which greater than 50% of the time points are deemed present and all the genes for which the present/absent calls exhibit a consistent and biologically relevant pattern (e.g., PAAA for the four time points in the experiment, indicating repression of expression of that gene over the course of the experiment). In all, we retain 5652 genes.

**Figure 1:**
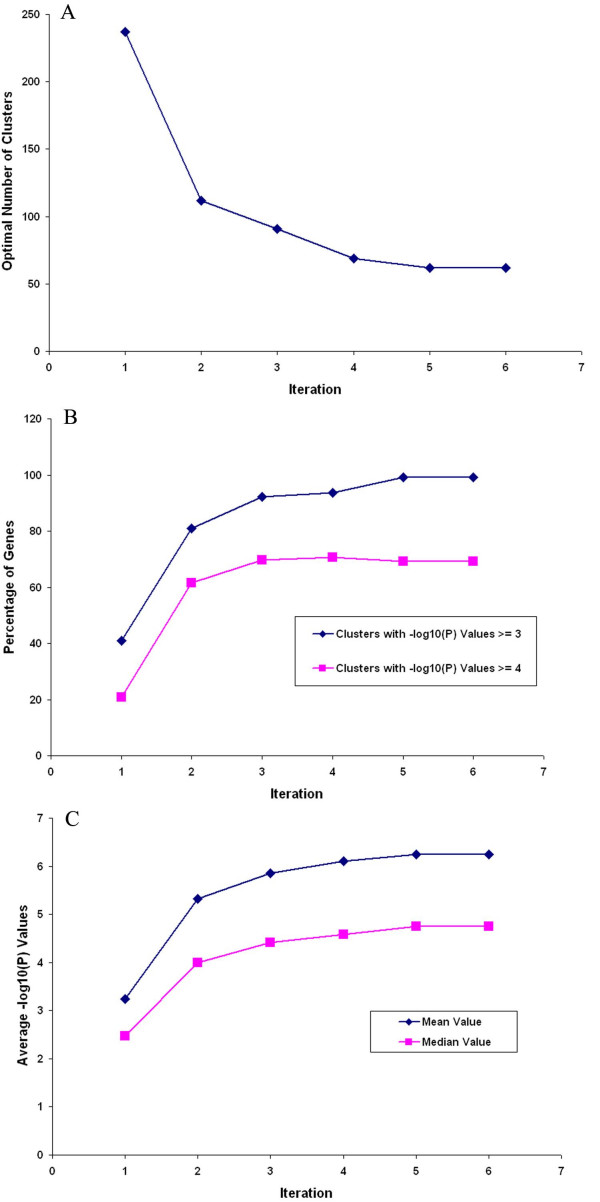
**Results of iterative clustering with dataset I**. A. Number of clusters as a function of the number of iterations of clustering dataset I with a p-value cutoff of 10^-4^. B. Percent of genes residing in biologically coherent clusters as a function of iteration cycle. Data are shown for the percent of clusters with a minimum of biological coherence of p-value less than 10^-3 ^and of p-values less than 10^-4^. C. Average p-value over the entire set of clusters as a function of iteration cycle.

Clustering dataset I using the EP_GOS_Clust algorithm described previously [4] results in 237 optimal clusters, out of which 46 were singleton clusters. We perform a GO analysis of the clusters and determined that 64% of the 5652 genes fall into clusters with significant functional coherence (p-values of 10^-3 ^or less; 32% of the genes fall into clusters with p-values of 10^-4 ^or less). Ignoring the singleton clusters, we also find that the average cluster size is 30.4, while the average size of clusters with p-values of 10^-3 ^or less is 36.2, thus indicating that clusters formed using the EP_GOS_Clust do not show strong biological coherence just because of a size bias. In fact, the average size of clusters with p-values of 10^-4 ^or less is only 36.9. We select as a cut-off standard a p-value of 10^-3^, which encompass 64 clusters containing a total of 2318 genes. Reclustering the remaining 3334 genes by another round of EP_GOS_Clust decreases the optimal number of clusters to 112, out of which 81% (or 4570 of the 5652 genes) of the genes fall into clusters with p-values of 10^-3 ^or less. After 6 iterations of this process, we find that the optimal number of clusters saturated at 62, with over 90% of the genes falling into clusters with p-values of 10^-3 ^or less. We also find that the proportion of genes that fall into clusters with p-values of 10^-4 ^or less increases from 32% to 69%. Figure 1 illustrates the results of the clustering process. As evident from this Figure, the proposed clustering method yields a monotonic increase in the average -log_10_(P) values and in the proportion of genes that found placement in quality coherent clusters, as well as a steady decrease in the optimal number of clusters, indicating the growing compactness and economy of the clustering. We further note that over 75% of the genes that were placed into singleton clusters for the first iteration remain in singleton clusters throughout suggesting that these genes are biological outliers. We also compare our clusters with that obtained using representative methods from the K-family of clustering approach [5-7], the Self-Organizing Map (SOM) [14], a variant that combines the SOM with hierarchical clustering, the Self-Organizing Tree Algorithm (SOTA) [15], and the QT-Clust method [13]. Table 1 shows that just the EP_GOS_Clust backbone already compares favorably against the other methods, and that the iterative approach further refines the cluster quality. In short, this iterative method clearly provides a means of enhancing the biological coherence of clusters obtained from microarray data.

**Table 1:** Comparison of biological coherence of clusters obtained for dataset I by different clustering algorithms

			Proportion of Genes (%) in Clusters of p-values	
			
		Average Correlation	<= 10^-4^	<= 10^-3^
(Clustering Method)	EP_GOS_Clust	0.617	32.8*	64.9*
	Iterated EP_GOS_Clust	0.685*	> 69*	> 90*
	KMedians	0.615	30.8	62.2*
	KCityBlk	0.398	27.5	56.7
	KCorr	0.630*	32.6*	60.1
	KMeans	0.614	25.1	55.2
	KAvePair	0.567	25.2	54.4
	QTClust	0.572	31.1	56.9
	SOTA	0.604	30.2	58.9
	SOM	0.623*	30.5	59.2

Shown are the comparative biological coherence formed by various clustering methods on dataset I. The 2 shaded rows represent clustering done by the standalone EP_GOS_Clust backbone and the proposed iterative approach.

* The top three performers in each category are indicated with an asterisk.

### Dataset II

We examine a second dataset derived from experiments designed to determine comprehensively the contribution of different signaling pathways to the glucose response in yeast. In addition to the Ras/PKA pathway, at least three other signaling pathways mediate transcriptional changes attendant on addition of glucose to cells [36,37]. In one pathway, glucose addition leads to reduced activity of the AMP-activated protein kinase encoded by *SNF1*, thereby unfettering a transcriptional repressor encoded by Mig1/2 and suppressing several transcriptional activators [38,39]. The second pathway, mediated by Rgt1, couples expression of the hexose transporter genes to the level of available glucose. Binding of glucose to plasma membrane glucose sensors promotes degradation of repressors of hexose transporter genes by the SCF^Grr1 ^ubiquitin ligase complex, allowing Rgt1 activation of these genes. Finally, the AGC kinase Sch9 acts in parallel to PKA to induce expression of many genes normally regulated by PKA [40]. To test the roles of each of these pathways, we measure expression changes following glucose induction in wild type and mutant cells lacking specific components of the different pathways. Levels of RNA for each of the 6237 yeast genes in each of the RNA samples are assayed using Agilent microarray chips, out of which measurable signals are registered from 5657 of them. This dataset consists of results from 23 time course experiments, each with 2–5 time points. [See additional file 5]

**Figure 2:**
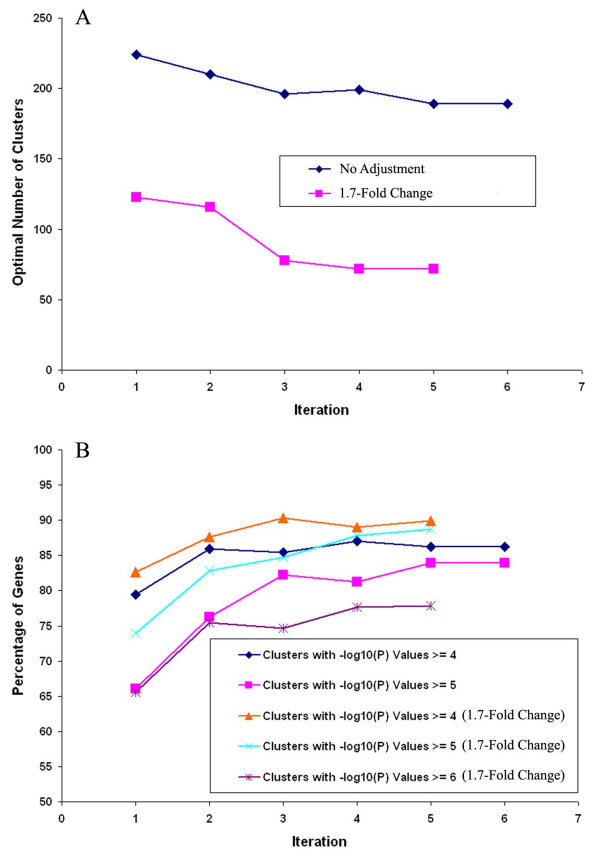
**Results of iterative clustering with dataset II**. A. Number of clusters as a function of the number of iterations of clustering dataset II with a p-value cutoff of 10^-4^, either including all 5657 genes in the Dataset (No Noise Adjustment) or including only those 4346 genes that exhibit a 1.7 fold change for at least 10% of the time points (1.7-Fold Change Noise Adjusted). B. Percent of genes residing in biologically coherent clusters as a function of iteration cycle. Data are shown for percent of clusters with a minimum of biological coherence of p-value less than 10^-4 ^and 10^-5 ^for all genes in the dataset and for a biological coherence of p-value less than 10^-4^, 10^-5 ^and 10^-6 ^for the subset of genes that exhibit a 1.7 fold change for at least 10% of the time points (minimal fold-change for meaningful clustering).

Clustering of dataset II using EP_GOS_Clust results in 224 optimal clusters, out of which 130 are singleton clusters. As before, we find the singleton clusters to be poorly related to the other genes, with a low average correlation coefficient of only 0.08. We perform a GO analysis of the clusters and note that 79% of the 5657 genes fall into clusters with p-values of 10^-4 ^or less and 66% of the genes fall into clusters with p-values of 10^-5 ^or less. This in itself already represents a highly robust quality of clustering. As a separate analysis, we ignore the singleton clusters and find that the average cluster size is 59.8, while the average size of clusters with p-values of 10^-4 ^or less is 78.2, thus indicating that clusters formed using the EP_GOS_Clust are unlikely to show strong biological coherence just because of a size bias. In fact, the average size of clusters with p-values of 10^-5 ^or less is only 81.1. For this dataset, we select as a cut-off standard a p-value of 10^-5^, which gives 44 clusters containing a total of 3737 genes. We apply the proposed methodology to improve the biological coherence of our clusters and found that after 6 iterations, the optimal number of clusters saturates at 189 (out of which 114 are singleton clusters), with 84% of the genes falling into clusters with p-values of 10^-5 ^or less (Figure 2, Table 2). We highlight that this is less than the final gene placement of over 90% obtained for dataset I for two main reasons. First, dataset II is drawn from a larger number of DNA microarray experiments and has 75 feature points compared to 24 for dataset I, thus introducing a greater range of variability within the data. Second, we imposed a stricter cut-off p-value of 10^-4 ^or less, as opposed to the previous choice of 10^-3^. Figure 2 summarizes the utility of our proposed methodology in improving cluster validity and placing the largest possible proportion of genes into biologically coherent clusters. We also find that the mean -log_10_(P) value of the clusters increases monotonically from 8.39 to 9.14.

**Table 2:** Improvement in biological coherence after iterative clustering of dataset II

	Proportion of Genes (%)			
	p-value <= 10^-4^	p-value <= 10^-5^	p-value <= 10^-4^	p-value <= 10^-5^
	(Uncorrected)		(Bonferroni-Corrected)	
Initial Iteration	79.4	66.1	57.2	52.2
Final Iteration	86.2	83.9	73.1	68.9

Shown are the percentages of genes in clusters in which one or more subsets of the genes in the cluster exhibit a statistically non-random membership in a common biological function, as defined by Gene Ontology (GO) classification, either at a significance level of 10^-4 ^or 10^-5^, either uncorrected (left) or Bonferroni corrected for multiple hypothesis testing (right). The values are for the clusters obtained following initial EP_GOS_Clust clustering or after six round of the iterative algorithm described in Methods.

At completion of the iterative process, we obtain 38 clusters with p-values of 10^-5 ^or less, containing 4747 (or 84%) of the original 5657 genes. We note that these clusters exhibit a tight grouping, as evidenced by a visual inspection of the gene expression time course plots [See additional files 1 and 2], as well as the relatively high values of correlation coefficients for these clusters (an average value of 0.667 over all clusters, with a maximum of 0.925 and a minimum of 0.387).

### Comparison of clustering methods for dataset II

We examine the coherence of the clusters obtained by our iterative version of EP_GOS_Clust versus the non-iterative version as well as other clustering methods. These include the well established K family of partition-based clustering algorithms [5-7], self organizing tree algorithms (SOTA) [15] and a recently proposed information theoretic-based method (IClust) [17]. As can be seen from the results in Table 3 the initial iteration of EP_GOS_Clust performs as well or better than any of these other methods, whether corrected for multiple testing or not, as measured both by the percent genes resident in clusters with high biological coherence and by the average expression correlation within individual clusters. Moreover, the clusters from the final iteration of the algorithm exhibit a higher level of expression correlation. Thus, the iterative EP_GOS_Clust compares favorably with other clustering methods.

**Table 3:** Comparison of biological coherence of clusters obtained for dataset II by different clustering algorithms

	Proportion of Genes (%) in Clusters of p-values			
	<= 10^-4^	<= 10^-5^	<= 10^-4^	<= 10^-5^
	(Uncorrected)		(Bonferroni-Corrected)	
EP_GOS_Clust	79.4*	66.1*	57.2	52.2*
Iterated EP_GOS_Clust	86.2*	83.9*	73.1*	68.9*
K-Means	78.0	62.1	58.0*	51.7
K-Correlation	77.1	63.9	57.3*	51.8
K-Medians	78.8*	65.3	56.9	52.2*
SOTA	75.2	66.9*	57.1	39.3
IClust	66.0	54.0	34.2	29.1

	Cluster Correlation			-log_10_(P) Values
	Max.	Min.	Ave.	Average

EP_GOS_Clust	0.920	0.454*	0.730*	9.17*
Iterated EP_GOS_Clust	0.956*	0.489*	0.750*	11.09*
K-Means	0.961*	0.049	0.668	9.01
K-Correlation	0.964*	0.398*	0.717*	9.13
K-Medians	0.923	0.203	0.683	9.09
SOTA	0.911	0.285	0.624	9.20*
IClust	N.A.	N.A.	N.A.	9.01

Methods include EP_GOS_Clust backbone, the iterative algorithm described in this report, the K-family of partitional clustering algorithms with pre-assigned clusters, self organizing tree algorithm (SOTA), and mutual information based clustering (IClust) [28]. Data in the upper table are presented as described in the legend to Table 2 while the lower table presents data on expression correlation within clusters and the average -log(P) values for biological coherence over all the clusters.

* The top three performers in each category are indicated with an asterisk.

### Function prediction based on expression profiles

An application of any clustering approach is the ability to predict the functions of unknown genes by clustering them together with counterparts with known functions. This is a particularly important consideration when working with organisms that are not as well-annotated as yeast. We test the capability of our proposed iterative procedure in this respect through a simulated study. We do this by randomly de-annotating 20% and 30% of the genes in dataset II. We then apply our iterative clustering approach to the entire dataset II and take into consideration the entire set of functional annotations reported on the SGD. Naturally at each iterative step, the clusters are scored as if the de-annotated genes have no known function, thus affecting the p-value. As a result, we find that our iterative procedure demonstrates a 61.5% level of prediction accuracy for the dataset with 20% de-annotation and 50.4% for the dataset with 30% de-annotation. We feel these results compare favorably to the other knowledge-based clustering methods reported in the literature [29,30]. These methods report a prediction accuracy of between 30–70%. However, these accuracies are found using restricted datasets of only 2–3 function classes (each containing 200–300 genes) and clustering is done up to 10–14 clusters. Furthermore, we observe there is very little variation in the percentage of prediction accuracy as our algorithm steps through the iterations. As a case in point, the prediction accuracy for the 20% test case in the 4 iterations it took for the optimal number of clusters to stabilize is 59.1%, 60.9%, 60.8%, and finally 61.5%. This and the relative high level of prediction accuracy by our iterative algorithm is a result of the clustering not being driven by the extent of known functional annotations, which can handicap the clustering process if the data is sparsely or wrongly annotated, but rather by fundamental indicators of cluster goodness. Hence, we expect the clustered results from a de-annotation simulated study or a GO permutation study to not severely affect the final results.

### Motif identification

Another means of evaluating the effectiveness of our clustering regimen is to determine the extent to which it reveals information regarding the underlying transcriptional network responsible for the observed pattern of transcription. For instance, are genes in specific clusters enriched for motifs corresponding to transcription factors known to be involved in regulation under the conditions tested? Several methods are available to identify motifs enriched in groups of genes and we applied a recent method – FIRE, which uses the concept of mutual information to predict functional motifs from gene expression data [41]. We compare the results obtained on this dataset clustered as described above with that from an expanded version of the same dataset clustered by K-means with a cutoff correlation of 0.85 [Zaman & Broach, unpublished]. Both sets of clusters reveal the role of the PAC/RRPE motif, sites for the transcription factors Msn2/4, Mbp1, Rap1 and Rpn4 as well as the 3' RNA binding factor Puf3. The K-means clusters also reveal a role for Gcn4, Hap4, Reb1 and Cbf1 while our iterative algorithm identifies Bas1 and Mig1. All these factors have been implicated in glucose regulation and the overlapping but non-identical results with the two cluster sets indicates the value of interrogating an expression dataset with multiple clustering methods.

### Abridged iterative approach

We examine the capabilities of an abridged version of our iterative algorithm. In the standard EP_GOS_Clust algorithm, we allow the formation of singleton clusters as a strategy to identify and isolate genes that are clear outliers. Mathematically, these singleton clusters have p-values of zero. Since it is reasonable to assume these genes play little or no part in the biological/cellular process of interest, having been put through a rigorous placement process, we drop these genes from future consideration instead of retaining them in the dataset for the iterative sorting process. As a result, the initial iteration on dataset II results in 224 optimal clusters. We then apply a p-value cut-off of 10^-5 ^or less as above, confirm the poor correlation of the singleton genes with the other genes, and then remove these from further consideration. We perform the same set of iterations and arrive at 118 as the final optimal number of clusters after only 3 rounds, out of which 31 are singleton clusters. Of these, 44 clusters have p-values of 10^-5 ^or less, as compared to 38 when using the full methodology. Nonetheless, Table 4 shows that the abridged method yields comparable results to the complete approach except in placing genes into clusters Bonferroni-corrected p-values of 10^-5 ^or less. Hence, as long as hypothesis testing errors are not a concern, the abridged proposal is valid and useful.

**Table 4:** Comparison of cluster coherence between the full and abridged versions of the iterative clustering algorithm

	Proportion of Genes (%) – Final Iteration			
	p-value <= 10^-4^	p-value <= 10^-5^	p-value <= 10^-4^	p-value <= 10^-5^
	(Uncorrected)		(Bonferroni-Corrected)	
Full Method	86.2	83.9	73.1	68.9
Abridged Method	86.3	84.5	72.7	65.1

Results from applying either the full iterative method or an abridged iterative method, as described in the text, to dataset II, executed with a threshold significance of 10^-5^.

Data are presented as described in the legend to Table 1.

### Minimal expression level for meaningful clustering

Gene expression measurements typically contain a certain amount of noise, which can come from hybridization errors due for instance to chip imperfections, as well as stochastic fluctuations in transcriptional processes. These are typically filtered by the appropriate microarray software, as described in the Methods section. As another area of interest, we then ask ourselves, noise aside, whether there can still be a minimal level of fold change for a particular gene to be considered relevant and can be meaningfully clustered.

We assume that for any particular gene's expression to be relevant, it should show a significant level of expression variation over at least 1–2 experiments. For dataset II, which contains 23 experiments, with 2–5 time points each, this corresponded to about 10% of the time points. Applying an arithmetic mean regression to this dataset yields a 1.7-fold change cut-off as the condition in which genes would demonstrate significant variation for at least 10% of all time points [See additional file 3]. From this, we derive a feasible region for fold-change cut-off by combining the constraint brackets of a time point allowance of at least 5–20% and the number of genes showing a reasonable level of intensity variation [See additional file 4]. We then cluster dataset II using several of these fold-change criteria candidates. Specifically, we parametrically test for (a) 1.9-fold change for at least 5% of time points – 4317 genes, (b) 1.8-fold change for at least 10% of time points – 4045 genes, (c) 1.7-fold change for at least 10% of time points – 4346 genes, and (d) 1.6-fold change for at least 15% of time points – 4280 genes. The results from this analysis are presented in Tables 5 and 6 and Figure 2.

**Table 5:** Effect of imposing minimal gene expression levels on cluster correlation

	No Adjustment	1.9-fold, at least 5% time points	1.8-fold, at least 10% time points	1.7-fold, at least 10% time points	1.6-fold, at least 20% time points
Number of Genes	5657	4317	4045	4346	4280
Optimal Clusters	224	135	118	123	140
Ave. Correlation	0.666	0.706	0.728	0.735	0.719
Max. Correlation	0.925	0.925	0.940	0.947	0.934
Min. Correlation	0.387	0.445	0.478	0.474	0.469

We compare the effects on genes retention and correlation results of within-cluster elements after clustering dataset II by the iterative algorithm.

**Table 6:** Effect of imposing minimal gene expression levels on biological coherence

	No Adjustment	1.9-fold, at least 5% time points	1.8-fold, at least 10% time points	1.7-fold, at least 10% time points	1.6-fold, at least 20% time points
	
	% of Genes in Clusters with -log_10_(P) Values Ranges				
> 0 and < 2	0.49	0.18	0.19	0.12	0.36
> 4	79.39	81.70	82.55	82.60	80.24
> 5	66.06	70.76	71.14	73.95	69.31

	Comparison of -log_10_(P) Values				

Mean Value	8.39	8.84	9.25	9.27	8.81
Best Value	59.82	66.67	70.96	69.44	66.23

We compare the effects on biological coherence of genes within clusters of dataset II obtained by the iterative algorithm.

The first observation from Tables 5 and 6 is that the results of clustering improve significantly after undertaking some form of gene expression relevance threshold. The second observation is that an over-strict and over-lenient fold-change threshold results in the respective deletion of useful information or the retention of non-information. As can be seen from Table 5, even though a cut-off criterion of 1.8-fold change for at least 10% of all time points results in the least number of genes and the best clustering economy in placing the genes into a lowest number optimal clusters, it does not result in the best overall correlation between members within the same clusters and clusters of the strongest biological coherence. On the other hand, the criterion of 1.7-fold change for at least 10% of the time points appear to produce the tightest clusters with the highest level of biological coherence despite having the largest number of genes as compared to the other fold-criteria tests. This is particularly noticeable from Table 6, where it places the largest proportion of genes into coherent clusters, but also has the smallest proportion of genes leftover for the weaker clusters. In addition, while this criterion does not lead to the lowest number of clusters, the resultant data groupings exhibit the strongest correlation. This leads us to conclude that a 1.7-fold cut-off for at least 10% is probably the appropriate screening criteria for dataset II.

We then assess the clusters using a newly-proposed functional genomics gold standard based on an expert curation of the Gene Ontology [42]. This allows us to assess our iterative process using only GO terms that are deemed specific enough to imply a meaningful biological relationship between any two annotated proteins. The iterative clustering of dataset II with a 1.7-fold cutoff gives a final 72 optimal clusters, of which 42 are quality clusters with p-values below the cut-off of 10^-5^. We performed a gold standard GO analysis of these 42 clusters and found that 41 of these returned meaningful annotation results based on this standard, and that the average precision and recall values of these clusters was 25.1% and 15.4% respectively (see Methods section). This significantly exceeds the average background of 1.8%. On the other hand, the average precision and recall values of the non-adjusted version of dataset II of 20.8% and 12.6% respectively, showing that the fold-change cutoff helps in improving the meaningfulness of the clustered results.

### Dataset III

Natural genetic variation can cause significant differences in gene expression, and clustering and linkage analysis can yield meaningful insights on how natural polymorphisms affect gene regulation. We test our iterative clustering approach on dataset III, which is obtained by measuring the expression levels of all yeast genes in a laboratory and a wild strain, and in 113 segregants from a cross between them [43,44]. The dataset consists of multiple replicates of parental strains and single arrays for each of the segregrants using spotted microarrays, yielding 131 experiments each with 7085 gene expression measurements. These genes correspond to 5740 known yeast genes, as well as 489 dubious ORFs and 856 control spots. [See additional file 5]

**Figure 3:**
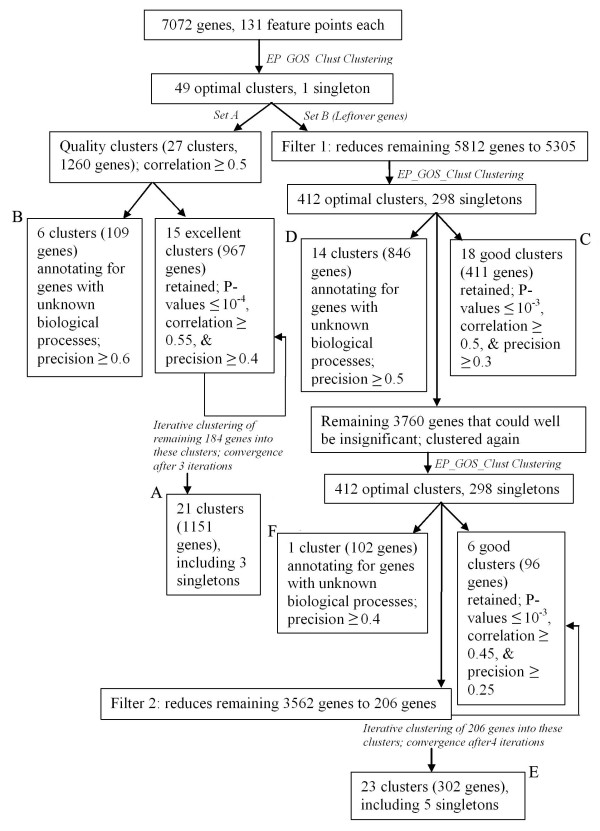
**Schematic showing the multi-stage clustering process for dataset III**. The full set of genes, including dubious ORFs and control samples are clustered by EP_GOS_Clust to yield 49 clusters. Those clusters with correlation ≥ 0.5 are retained and split into two groups. Those with ≥ 60% of their member genes annotated as unknown biological function are set aside as group B. The second group is subjected to iterative clustering as described in Methods, with a threshold p-value of 10^-4^, yielding 21 clusters (group A). The remaining genes from the initial clustering process are first filtered to remove those with little correlation to any other gene or limited expression. Those genes passing the filter are subjected to EP_GOS_Clust and those clusters exhibiting expression correlation ≥ 0.5 are examined. Those clusters that also have at least 30% their genes annotated to a common function with a p-value less than 10^-3 ^are retained as group C. Those with ≥ 50% of their member genes annotated as unknown biological function are set aside as group D. The remaining genes are once again clustered by EP_GOS_Clust, yielding one cluster with ≥ 40% of their member genes annotated as unknown biological function (group F) and several clusters with the indicated correlation, precision and coherence. The remaining 3,760 genes are then stringently filtered. Since the genes have already been subjected to clustering, we can assume that the most useful information has already been sieved out. The remaining 3562 genes are probably all irrelevant, but we would still like to identify the genes that have significant levels of expression. We hence look at the number of genes that has a minimum proportion of feature points falling within the data mean ± 0.5*(standard deviation), and find that as the pre-determined proportion is decreased, the number of genes increases almost linearly until the 77% mark, where it then starts to grow exponentially. We take this to signify an increasing bulk of spurious genes and set the cut-off at 77% to extract 206 genes for further clustering. This yields the final group of clusters (group E).

Unlike the previous two datasets, which were obtained under controlled experimental conditions, dataset III is a much more diverse set of expression conditions, rendering it more challenging to cluster. Clustering dataset III using EP_GOS_Clust results in 49 optimal clusters. We note that even at this level, the cluster correlation and biological coherence compares favorably against previous hierarchical clustering results [44], which for a minimum pair-wise correlation of 0.725 yields 763 clusters of at least two genes containing a total of only 2522 genes out of the original 7085. As a test, we first adopt a multi-stage approach with progressive filtering to cluster it. The rationale for this approach is that successively extracting the higher correlated clusters allows subsequent clustering in the absence of these strong attractors. The scheme we follow and the results of that process are outlined in Figure 3.

The process of sequential clustering allows us to identify a total of 1864 genes in 62 quality clusters (indicated by A, C and E) that annotates for known biological functions, as well as 21 clusters (indicated by B, D and F) that contain 1057 genes of unknown biological processes. This compares favorably both in coverage and compactness with the 2544 genes sorted into 763 clusters by the hierarchical clustering method previously applied to this dataset. Furthermore, application of our iterative clustering approach at two instances (A and E) allowed us to improve cluster quality, in terms of member correlation, cluster precision, and p-value. The iterative approach also improves the number of genes placed into good clusters. For instance, for group A, the number of genes falling within clusters with p-values of 10^-6 ^and below improves from 986 to 1084, while for group E, the number goes from 85 to 164.

**Figure 4:**
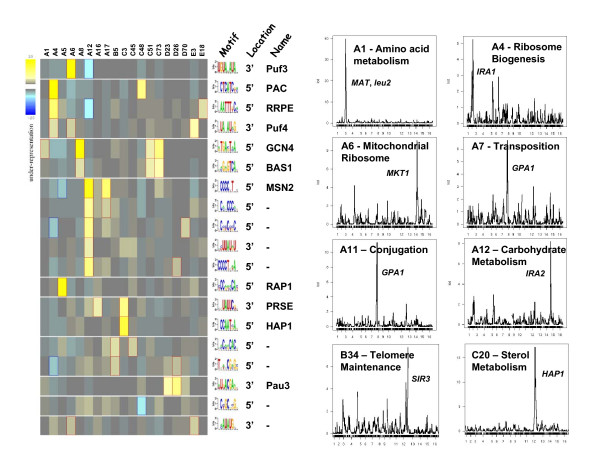
**Functional analysis of dataset III clusters**. Genes in clusters obtained as described in Figure 3 are assessed for motif enrichment in their 5' and 3' flanking regions using FIRE. On the left is the subject of clusters (columns) exhibiting statistically significant enrichment (shades of yellow) or exclusion (shades of blue) of a motif (rows) whose consensus sequence is shown to the right. If known, the name of the motif or the factor that likely binds to it is provided. The clusters are also examined to determine whether the expression pattern of genes in a clustered are associated with a gene segregating in the cross from which the data is derived. The LOD (log of the odds) score of linkage to each 20 Kb bin across the entire yeast genome is shown for a representative subset of the clusters. A potential trans-acting factor encoded within the interval of the elevated LOD score is shown next to the peak.

We evaluate the clustered results in two ways. First, as described for the previous dataset we apply FIRE to extract regulatory motifs associated with individual clusters. Second, we applied linkage analysis to the clustered expression data to determine whether the pattern of expression in a particular cluster could be associated with an unlinked site in the genome. This process identifies genes whose product would likely be acting in trans to modulate expression of genes in a particular cluster [43,44]. The results of these analyses are presented in Figure 4 and summarized in Table 7. As evident from this summary, the serial clustering approach yields highly coherent clusters, both in expression correlation and in biological function, even in the third round of clustering (group E). Moreover, analysis of the genes clustered by this method identifies most of the cis-acting motifs and the trans-active sites that had been extracted from this dataset previously. In addition, the FIRE analysis of the clustered data identifies a number of enriched motifs in the 3' sequence of genes, which have not been extracted previously from this dataset but which correspond to a number of previously noted motifs associated with mRNA stability, including Puf3, Puf4 and PRSE, as well as a motif associated with the PAU gene family of unknown function [45]. As previously recognized, the trans-acting factor(s) that affect(s) expression of genes in a cluster generally does not correspond to the transcription factor(s) associated with the motifs enriched in genes in that cluster. Rather, these trans-acting factors predominantly define physiological processes – mating type (*MAT*) or prototrophy (*leu2 *versus *LEU2*), for example – or signaling networks – Ira1, Ira2, Ras1 or Gpa2 – that impinge on the expression patterns indirectly. In this context, though, it is intriguing to note that the same allele variation in a trans-acting can induce multiple, distinct expression patterns, witnessed by the fact that the same locus is linked to several different clusters. This would suggest that the factor may work in combination with other loci segregating in the cross to yield a variety of transcriptional patterns. Such polygenic effects are expected but have only begun to be rigorously explored.

**Table 7:** Summary of clustering results with dataset III

Cluster Group		Cluster Size		-log_10_(P) Values			Correction	Correlation	Precision
		Max.	Min.	Ave.	Max.	Min.	Ave.	Ave.	Ave.
	B	61	4	2.5	4.2	1.2	1.7	0.609	0.753
	D	175	8	5.5	13.7	1.5	3.9	0.362	0.641
	F	102	102	2.9	2.9	2.9	0.9	0.172	0.494
A	Initial	271	2	20.0	140.1	0^a^	19.7	0.655	0.461
	Final	271	2	21.7	140.1	1.8^b^	20.6	0.707	0.522
C		116	2	10.4	33.3	3.2	9.5	0.672	0.735
E	Initial^c^	-	-	-	-	-	-	-	-
	Final	88	2	4.4	11.4	1.1	3.4	0.635	0.440

Genes in dataset III are clustered by EP_GOS_Clust through a sequential process outlined in Figure 3. Genes in cluster groups A and E are further clustered by the iterative algorithm, yielding an initial and final set of clusters. Precision is defined as the fraction of genes within a cluster assigned to the predominant functional group within that cluster.

^*a*^*The cluster p-value is zero if a GO search did not manage to uncover any significant annotation*.

^*b*^*After iteratively clustering 184 genes into 15 initial clusters ('A' on Figure 3), just one poor cluster remains. The next worse cluster has a -log*_10_*(P) value of 4.1*.

^*c*^*There are no applicable initial values here since the remaining genes to be clustered are subjected to the second filter before being re-clustered into the initial 6 clusters (see Figure 3)*.

Next, as a test of our iterative procedure, we subject dataset III to this algorithm without any intermediate filtering or multi-stage processing. From the initial clusters obtained from the first round of clustering, the iterative procedure results in 106 optimal clusters after 6 iterations, based on a p-value cutoff of 10^-5^. A subsequent analysis of the clusters reveals results comparable to that obtained from the application of intermediate filtering as described previously. The large clusters that annotate for major functions such as translation, ribosome biogenesis, cellular component organization, and chromatin assembly appear in both instances, and have about 60% of their member genes in common. The smaller clusters that annotate for flocculation, cytokinesis, vitamin B6 metabolism, RNA-mediated transposition, and sterol biosynthesis far even better, with 75% of common genes recovered in both cases. The average specificity of the clusters obtained by using just the iterated procedure is 33%, which compares well with the 25–75% specificity range (average of 52%) obtained earlier. We also note the uncovering of several clusters whose majority of genes have unknown biological processes. In both applications, the cluster specificity is high – an average of 33% compared to a range of 40–60% by using multi-stage clustering with intermediate filters. In addition, the number of clusters obtained (i.e, 106) is similar to the total of 89 quality clusters retained by the previous application. We note however that the optimal clusters obtained by using just the iterated approach without any filters is lower – an average of 0.497 versus 0.656. This and the comparatively lower cluster specificity is understandably the result of the two filters implemented in the former process, including one that involves correlation filtering. Nonetheless, the recovery of several common-function clusters with overlapping genes leads us to believe that our iterative approach would be effective in uncovering clusters with a strong level of biological coherence even without applying various levels of additional filtering to a dataset.

## Conclusion

Uncovering biological insights from DNA microarray data is a promising but challenging task. Generally, the first step in organizing and analyzing microarray data is clustering genes into groups related by expression patterns, which often then reveals biological coherence. In our study, we observe that the task of placing genes into strongly coherent clusters may not necessarily be achieved with a single round of clustering, no matter how robust the clustering algorithm has proven to be. To address this limitation we formulated a methodology that filters out the genes placed in clusters of weak biological coherence and iteratively seeks the best placements for these genes. We show that on the whole our proposed algorithm unambiguously refines the biological quality of gene clusters. The extent of improvement over the iterations is significant, from a 20% increase in the number of genes in biologically coherent clusters for dataset III to a 40% increase for dataset I. We note also the increasing level of average cluster correlation as the iterative sorting progresses. The latter observation is significant, since cluster correlation is not a factor explicitly used in the algorithm to target clusters for recycling.

We apply this algorithm to a variety of datasets. Two of these datasets are strongly focused on the question of nutritional regulation in yeast. We select the third dataset as a test for this method since, due to experimental design, it is as diverse a collection of expression patterns as any in the literature (Myers C, personal communication). We evaluate our results from the second dataset against those obtained by a number of standard clustering algorithms and find that our method assigns a higher proportion of genes to biologically coherent clusters than any of the others. Moreover, the average expression correlation of genes in the clusters is higher by our method than by any of the others. In addition, subsequent analysis of the clusters reveals a strongly overlapping set of predictions for cis-acting regulatory sequences to that obtained from clusters generated by other methods. Our results with the third dataset also compare favorably with other methods. Our algorithm, applied in a multi-stage fashion, assigns 2921 genes to 83 clusters compared with 2544 genes in 763 clusters previously compiled by hierarchical clustering with a correlation value cutoff. This reduced number of clusters facilitates subsequent analysis of the data while retaining essentially all of the information content, as assessed by analysis of linkage to trans-acting factors and identification of cis-acting regulatory domains.

An issue with incorporating annotative knowledge is that the clustering process can be limited when applied to datasets from sparsely or inaccurately annotated organisms. In this respect, our iterative procedure still performs comparatively well under these situations. On its own, the clustering backbone EP_GOS_Clust compares favorably against several well-known clustering algorithms based on not just the usual quantitative attributes but also the level of biological coherence based on GO resources. The procedure has as its pre-processing step a complete clique search (itself a rigorous search process) amongst the data points for strong pre-clusters. In our iterative procedure, GO knowledge is not considered during the clustering itself, but rather in post-processing and cluster refinement steps. The clustering backbone thus considers only intuitive quantitative attributes, so a lack of functional annotation does not affect the result as adversely as other more involved knowledge-based clustering approaches. The iterations then smooth any distortions brought about by issues with existing annotation databases or measurement inconsistencies.

Based on the results of this study, we believe our work to be valuable in uncovering biologically meaningful data structures. Since the complex functions of a living cell are carried out through a concerted activity of many gene and gene products, it is important to be able to effectively cluster DNA microarray data to uncover functional relationships and regulatory modules to help understand the complex biological mechanisms involved in signaling.

## Methods

### Notation

We denote the distance measure of a gene i, for i = 1,....,n with k features (or dimensions), for k = 1,....., s as a_ik_. Each gene expression pattern is transformed into a k-dimensional vector, for which each element indicates the change in normalized expression level between time points for each gene, a_ik_. Each gene is assigned to only one (hard clustering) of c possible clusters, each with center z_jk_, for j = 1,....,c. The binary variables w_ij _indicates whether gene i falls within cluster j (w_ij _= 1, if yes; w_ij _= 0, if no).

### Microarray data processing

The experiments for datasets II and III are carried out using the Agilent microarray platform. All arrays are hybridized and processed using a SSPE wash according to the manufacturer's protocols, and the microarrays are imaged using a Agilent microarray scanner. The images are then extracted with Agilent Feature Extraction version A7.5.1 and the data analyzed with Rosetta Luminator 2.0 gene expression analysis system (Rosetta Informatics, Seattle, WA). Using a rank consistency filter, features are subjected to a combination linear and LOWESS normalization algorithm, the recommended algorithm for this microarray platform. The Agilent microarray scans feed directly into the Princeton University Microarray Database (PUMAdb) [46], where the noise filtering is performed in tandem. PUMAdb is based on the Stanford Microarray Database software package [47]. The filtering process involves identifying the Cy3 and Cy5 intensities above the background levels and picked out features that were either population outliers or non-uniformity outliers.

### Handling missing data points

DNA microarray results often contain missing data points for a variety of reasons. In our study, dataset II contains about 0.4% of missing data points. To address this, we examine the row average of the unblemished data points and apply a variant of the K-Nearest Neighbor algorithm to estimate the missing points [48]. The procedure we use is as follows:

(i) For a particular gene with missing data points, we calculate its level of similarity with all other genes that have values present for the particular data points. The similarity metric is consistently computed by excluding the data column containing the missing data point. In this respect, we test both the Euclidean distance and Pearson Correlation Coefficient as measures of data similarity and find that both work similarly well in estimating the 'removed' data points.

(ii) For each gene with missing data points, we create a rank-order list of its similarity with its reference genes. Depending on the spread of similarity level, we estimate the missing data point by taking the sum average of its nearest 10–20 neighbors.

### Normalization and pre-clustering

Pre-clustering the data helps minimize the computational resources required to solve the hard clustering problem by (i) identifying genes with similar experimental responses, and (ii) removing outliers deemed not to be significant to the clustering process. A straightforward pre-clustering approach to provide just the adequate amount of discriminatory characteristics so that the genes can be pre-clustered properly is to reduce the quantities represented in the k-dimensional expression vectors into a set of representative variables [+, o, -]. The (+) variable represents an increase in expression level compared to the previous time point, the (-) variable represents a decrease in expression level from the previous time point, and the (o) variable represents an expression level that does not vary significantly across the time points (which we take for the purpose of this study as a range of ± 10% from the zero normalized value). We can also pre-cluster the expression data by creating a rank-ordered list of gene proximities based on Euclidean distance or correlation. We then group genes that demonstrate an obvious level of proximity, such as a separation of only at most 1% of the maximum inter-gene distances. Here, we choose to pre-cluster the proximity genes that form a complete clique. This means the pre-clustered genes that belong uniquely to only one cluster, or in other words, there is a link between every gene within the same cluster. With this choice, we can perform a maximal clique search by using various levels of pre-clustering criteria. In pre-clustering over a range of cut-off values, we are able to select the appropriate criterion as the point where the maximum number of complete cliques is formed [49]. In this study, we first z-normalize the expression data across each gene and then compute the feature vector (that is, the change in expression level between time points). The normalization reduces expression data to a standard normal N(0,1) according to:

$$a_{ik}^{normalized}=\frac{a_{ik}^{unnormalized}-E_{i}\left(a_{ik}\right)}{\sigma_{i}\left(a_{ik}\right)},\forall i{\text{, where E}}_{\text{i}}\left(a_{ik}\right)=\frac{1}{s}\sum_{k=1}^{s}a_{ik}\text{ and }\sigma_{\text{i}}\left(a_{ik}\right)=\sqrt{E_{i}\left(a_{ik}^{2}\right)-{\left(E_{i}\left(a_{ik}\right)\right)}^{2}}$$

### EP_GOS_Clust clustering algorithm

We perform EP_GOS_Clust clustering by the method described in detail in [4].

The global optimization approach seeks to minimize the sum of the Euclidean distances between the data and the assigned cluster center. There are two sets of variables in the problem, w_ij _and z_jk_. While the bounds of w_ij _are clearly 0 and 1, that of z_jk _is obtained by observing the range of a_ik _values.

$$\begin{array}{l} z_{jk}^{L}=\min\left\{a_{ik}\right\},\forall k=1,.....,s\;\\ {\text{z}}_{\text{jk}}^{\text{U}}=\max\left\{a_{ik}\right\},\;\forall \text{k}=\text{1,}.....\text{, s} \end{array}$$

The basic clustering problem is:

(1.1) $$\begin{array}{ll} \underset{{\text{w}}_{\text{ij}},z_{jk}}{\text{Minimize}}\; & \sum_{\text{i}=\text{1}}^{\text{n}}\sum_{j=1}^{c}\sum_{k=1}^{s}w_{ij}{\left(a_{ik}-z_{jk}\right)}^{2}\\ \text{s}\text{.t}\text{.} & \sum_{\text{j}=\text{1}}^{\text{c}}w_{ij}=1,\;\forall \text{i}=\text{1,}.....\text{, n} \end{array}$$

*w*_*ij *_are binary variables, z_jk _are continuous variables

The proximity study in pre-clustering will determine the spread of clusters each gene is suitable for. This can be described by an additional binary parameter suit_ij_. A data point that has been determined to belong uniquely to just one cluster in the pre-clustering process will only have suit_ij _= 1 for only one value of j and zero for the others, whereas a data point restricted to a few clusters will have suit_ij _= 1 for only those clusters. The suit_ij _parameters obviate the need for constraints that prevent the redundant re-indexing of clusters and help reduce the computational demand required for the clustering process.

The objective function in Problem 1.1, when expanded is:

$$\sum_{j=1}^{c}w_{ij}\sum_{i=1}^{n}\sum_{k=1}^{s}a_{ik}^{2}-\sum_{i=1}^{n}\sum_{j=1}^{c}\sum_{k=1}^{s}a_{ik}w_{ij}z_{jk}+\sum_{j=1}^{c}\sum_{k=1}^{s}z_{jk}\sum_{i=1}^{n}w_{ij}\left(z_{jk}-a_{ik}\right)$$

Together with the necessary first-order optimality condition:

$$\sum_{i=1}^{n}w_{ij}\left(z_{jk}-a_{ik}\right)=0,\;\forall j\text{, }\forall \text{k}$$

(i.e., the vector distance sum of all genes within a cluster to the cluster center should be intuitively zero), and the constraint that each gene is allowed to belong to only one cluster, (i.e. $\sum_{j=1}^{c}w_{ij}=1$), the formulation becomes:

(1.2) $$\begin{array}{ll} \underset{{\text{w}}_{\text{ij}},z_{jk}}{\text{Minimize}} & \sum_{\text{i}=\text{1}}^{\text{n}}\sum_{k=1}^{s}a_{ik}^{2}-\sum_{i=1}^{n}\sum_{j=1}^{c}\sum_{k=1}^{s}\left(suit_{ij})(a_{ik}w_{ij}z_{jk}\right)\\ \text{s}\text{.t}\text{.} & {\text{(suit}}_{\text{ij}}{\text{)(z}}_{\text{jk}}\sum_{i=1}^{n}w_{ij}-\sum_{i=1}^{n}a_{ik}w_{ij})=0,\;\forall \text{j, }\forall \text{k}\\ & \sum_{\text{j}=\text{1}}^{\text{c}}(suit_{ij})w_{ij}=1,\;\forall \text{i}\\ & \text{1}\leq \sum_{\text{j}=\text{1}}^{\text{n}}(suit_{ij})w_{ij}\leq n-c+1\\ & {\text{w}}_{\text{ij}}=\text{0-1,}\forall \text{i,}\forall \text{j}\\ & {\text{z}}_{\text{jk}}^{\text{L}}\leq {\text{z}}_{\text{jk}}\leq {\text{z}}_{\text{jk}}^{\text{U}}\text{,}\forall \text{j,}\forall \text{k} \end{array}$$

The first set of constraints are the necessary optimality conditions, the second demand that each gene can belong to only one cluster, and the third state that there is at least one and no more than (n-c+1) data points in a cluster. Note also that the $\sum_{i=1}^{n}\sum_{k=1}^{s}a_{ik}^{2}$ term in the objective function of Problem 2 is a constant and can be dropped, though for the sake of completeness we will retain the term throughout.

Problem 1.2 is a Mixed Integer Nonlinear Programming (MINLP) problem with bilinear terms *w*_*ij*_.*z*_*jk *_in the objective function and the first constraint set. However, MINLP problems are difficult to solve and theoretical advances and prominent algorithms for approaching such problems have been extensively studied. We utilize the Global Optimum Search (GOS) algorithm [31-33] to handle the MINLP formulation. The algorithm decomposes the nonlinear problem into a primal problem and the master problem. The former optimizes the continuous variables while fixing the integer variables and provides an upper bound solution, while the latter optimizes the integer variables while fixing the continuous variables and provides a lower bound solution. The two sequences of upper and lower bounds are iteratively updated until they converge in a finite number of iterations. Even though the algorithm does not have a theoretical guarantee of finding the global optima, its application in a robust clustering procedure has been shown to perform favorably against existing clustering methods when applied to DNA microarray data [31].

The primal problem results from fixing the binary variables to a particular 0–1 combination. Here, w_ij _is fixed and z_jk _is solved from the resultant linear programming (LP) problem. The primal problem is given by:

(2.1) $$\begin{array}{ll} \underset{{\text{z}}_{\text{jk}}}{\text{Minimize}} & \sum_{\text{i}=\text{1}}^{\text{n}}\sum_{k=1}^{s}a_{ik}^{2}-\sum_{i=1}^{n}\sum_{j=1}^{c}\sum_{k=1}^{s}a_{ik}w_{ij}^{*}z_{jk}\\ \text{s}\text{.t}\text{.} & {\text{z}}_{\text{jk}}\sum_{i=1}^{n}w_{ij}^{*}-\sum_{i=1}^{n}a_{ik}w_{ij}^{*}=0,\;\forall \text{j, }\forall \text{k} \end{array}$$

In Problem 2.1, all the other constraints drop out since they do not involve z_jk_, the variables to be solved in the primal problem. Besides z_jk_, the Lagrange multipliers λ_jk_^m ^for each of the constraints above is obtained. The objective function is the upper bound solution. These go into the master problem. The master problem is essentially the problem projected onto the y-space (i.e., that of the binary variables). To expedite the solution of this projection, the dual representation of the master is used. This dual representation is in terms of the supporting Lagrange functions of the projected problem. Also, the optimal solution of the primal problem as well as its Lagrange multipliers can be used for the determination of the support function, which are gradually built up over each successive iteration. The master problem is:

(2.2) $$\begin{array}{l} \underset{w_{ij},\mu_{B}}{\text{Min}}\mu_{B}\\ \text{s}\text{.t}\text{. }\mu_{B}\geq \sum_{\text{i}=\text{1}}^{\text{n}}\sum_{k=1}^{s}a_{ik}^{2}-\sum_{i=1}^{n}\sum_{j=1}^{c}\sum_{k=1}^{s}a_{ik}w_{ij}z_{jk}^{*}+...\\ \;...\sum_{j=1}^{c}\sum_{\text{k}=\text{1}}^{\text{s}}\lambda_{jk}^{m*}({\text{z}}_{\text{jk}}^{\text{*}}\sum_{i=1}^{n}w_{ij}-\sum_{i=1}^{n}a_{ik}w_{ij}),m=1,M\\ \;\sum_{\text{j}=\text{1}}^{\text{c}}w_{ij}=1,\;\forall \text{i}\\ \text{ 1}\leq \sum_{\text{j}=\text{1}}^{\text{n}}w_{ij}\leq n-c+1,\forall j \end{array}$$

The master problem, which is a Mixed Integer Linear Programming (MILP) problem solves for w_ij _and μ_B_, and gives a lower bound solution in the objective function. The w_ij _solutions then go back into the primal and the process is repeated. A new support function is added to the list of constraints for the master problem with each iteration. Thus in a sense, the support functions for the master problem build up with each iteration, forming a progressively tighter envelope and gradually pushing up the lower bound solution until it converges with the upper bound solution. In addition, after every run of the master problem, in which a solution set for w_ij _is generated, an integer cut is added for subsequent iterations to prevent redundantly considering that particular solution set again. The cut is expressed as:

$$\sum_{i\in \left\{n|w_{ij}=1\right\}}^{n}w_{ij}-\sum_{i\in \left\{n|w_{ij}=0\right\}}^{n}w_{ij}\leq n-1$$

In implementing the algorithm we let the initial clusters be defined by a robust set of pre-clustered genes and compute the centers of these clusters. As a good initialization point we place the remaining genes into the nearest cluster. A proximity study assesses each gene's suitability in an appropriate number of clusters. After the initial GOS [31-33] run, the worst placed gene is removed and used as a seed for a new cluster. This gene has already been subjected to the initial search for parameters so there is no reason for it to belong to any one of the older clusters. Based on the updated centers, the iterative steps are repeated. With each GOS [31-33] cycle, the number of clusters builds up progressively. At each cluster number, we compute the clustering balance, which is the α-weighted sum of the intra-cluster and inter-cluster error sums. The clustering balance parameter reaches a minimum at the optimum cluster number, when intra-cluster similarity is maximized and inter-cluster similarity is minimized. Hence, the incrementing of the cluster number can be stopped once this turning point is reached.

### Biological coherence assessment and refinement

**Figure 5:**
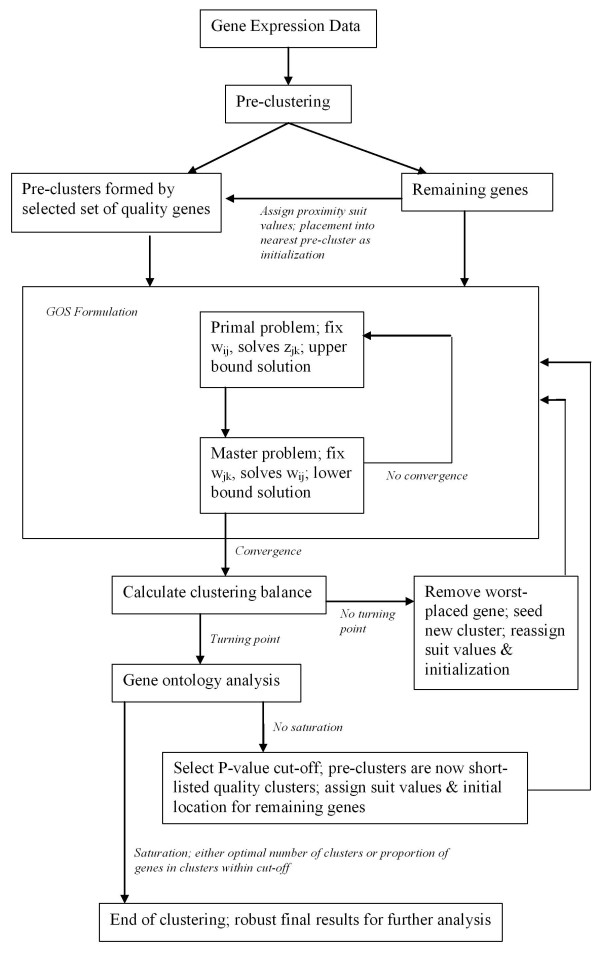
**Schematic of the EP_GOS_Clust clustering algorithm used in this study**. Although the formulation in this study has been notated for DNA microarray data, the algorithm framework can be adapted for clustering any numeric data.

We determine the biological coherence of each cluster by assessing whether it contained genes assigned to a common biological function, as specified by Gene Ontology (GO) annotations available through the Saccharomyces Genome Database [50]. This is quantified as the likelihood that the subset of genes in that cluster with a common biological function could have been assigned to that cluster by chance (p-value). Since a single full run of the EP_GOS_Clust does not necessarily assign a number of genes to clusters of high biological coherence, we employ an iterative approach to clustering by imposing a coherence floor based on p-values to demarcate genes that have already been well clustered. As described by the original EP_GOS_Clust algorithm, we take these quality clusters as the initial set of clusters. We then compute the Euclidean distance between each of the remaining genes (coming from clusters with low biological coherence) and these initial centers and as a good initialization point placed these genes into the nearest cluster based on $Min\left\{\sum_{k=1}^{s}{\left(a_{ik}-z_{jk}^{initial}\right)}^{2},\forall j\right\}$. We create a rank-order list for each of these remaining genes for its distance to each of the initial clusters, and for each gene allow its suitability in the nearest clusters via suit_ij _parameters. The initialization point and suit_ij _parameter assignments are then utilized in the GOS algorithm all over again. Through these iterations, we offer the poorly-placed genes an opportunity either to find relevant membership in one of the strongly coherent clusters or to regroup among themselves to form quality clusters. We have found that reiteration of this process eventually yields a saturation point whereby the optimal number of clusters becomes constant of the proportion of genes distributed within clusters of high biological coherence levels off. This algorithm can be summarized by the schematic in Figure 5.

### Calculating cluster p-value

The p-value of each cluster is a measure of the statistical significance for functional category enrichment. The biological coherence of each cluster is scored according to the percentage of its genes covered by annotations significantly enriched in the cluster in question, and is computed using a hypergeometric distribution. If G is the number of genes annotated to a term and N is the total number of genes in the genome with GO annotations, then the probability *p *of a randomly selected gene being annotated to a particular GO term is GN. Given a cluster of n genes, in which × of them have been annotated to a given GO term, the probability of having × out of n annotations assigned to the same GO term by chance is *p*^*x*^(1 - *p*)^*n*-*x*^. Within a list of n genes, there are multiple permutations by which × of them have this annotation. The number of permutations is n!x!(n−x)!. However, annotations to a particular term are low probability events. Thus instead of calculating the probability of having × of n genes annotated to a term, a more conservative approach is to calculate the probability of × or more of n genes being annotated to a particular term. This is given as ∑j=xn(n!j!(n−j)!)pj(1−p)n−j. In analyzing the gene clusters, we consider the p-value of the most significant term associated with each cluster.

Sometimes, a cut-off for the p-value, known as alpha, is chosen, such that p-values below the alpha are deemed significant. For instance, an alpha level of 0.05 means that in no more than one in 20 statistical tests will the test show 'something' while there is in fact nothing (a type I error). However, when more than one statistical test is carried out, there is an increasing chance of committing a type I error due to fluctuations. This chance is given by 1-(1 - *α*)^*k*^, where k is the number of tests (i.e. the number of elements in a cluster). A possible solution known as the Bonferroni correction is to divide the test-wise significance level by the number of tests. For instance, given an initial alpha of 0.05, the chance of a type I error if 10 tests are made is 0.4. The Bonferroni correction adjusts the alpha so the overall risk for the tests remains at 0.05, by applying a significance level of 0.005 instead of 0.05. We include both the p-values provided by the SGD and our Bonferroni-corrected values in our manuscript for the sake of completeness.

### Filtering dataset III

The first filter applied to dataset III eliminates those genes for which 75% of the values were zero, after background subtraction, or that show a correlation of less than 0.1 with any other gene. The second filter involves computing the expression mean and standard deviation over all segregants (feature points) for all of the 3,760 genes that remain after an initial clustering of Set B data (see Figure 3), which yields a normal distribution. We then determine for each gene what proportion of feature points had values falling outside the data mean ± 0.5 (standard deviation). Plotting the cumulative number of genes with a given proportion of feature points that lie outside this limit versus proportion of feature points, we find that as the proportion of feature points decreased from 100%, the number of genes increases almost linearly until the 77% mark, at which point the number of genes increases essentially exponentially. We take this to signify an increasing bulk of spurious genes and set the cut-off at 77% retained those 206 genes with values between 77% and 100% for further clustering.

### Linkage analysis

Linkage analysis is performed on the average phenotype of each cluster. After mean centering each transcript, the average value of all transcript levels within a cluster is calculated and treated as a quantitative phenotype as previously described [44]. Linkage analysis is then performed using the nonparametric test in R/qtl [51] with 2894 previously described markers [43]. Empirical permutation tests [52] are used to determine a false discovery rate (FDR), with a LOD score of 3.4 estimated as a cutoff for a 5% false discovery rate.

### 'Gold standard' evaluation of functional genomic data

Rigorous analysis of gene clusters for functional annotations requires a quality collection of genomic functional categories. [42] propose a unified standard based on expert curation so that only functional terms that are specific enough to be confirmed or refuted experimentally and yet general enough to provide relevant information for high-throughput assays are considered. The evaluation framework and gold standard can be found at [53]. As performance measures, we use the recall, which is the ratio of the number of relevant records retrieved to the total number of relevant records in the database, and the precision, which is the ratio of the number of relevant records retrieved to the total number of irrelevant and relevant records retrieved. Given the number of true positives (TP) – protein pairs associated by data and annotated as positives in the gold standard, false positives (FP) – protein pairs associated by data and annotated as negatives in the gold standard, and false negatives (FN) – protein pairs not associated by data and annotated as positives in the gold standard, for each cluster, the metrics are given by:

$$\begin{array}{l} \text{Precision}=\frac{TP}{TP+FP}\equiv \frac{\text{Number of genes annotated for a particular function}}{\text{Number of genes in the cluster}}\\ \text{Recall}=\frac{\text{TP}}{\text{TP}+\text{FN}}\equiv \frac{\text{Number of genes annotated for a particular function}}{\text{Genomic frequency for the particular function}} \end{array}$$

In addition, we introduce as a minimal benchmark of gold standard validation the background, given by:

$$\text{Background}=\frac{\text{Genomic frequency for a particular function}}{\text{Genomic Size}}$$

This is the minimal benchmark as it signifies the probability of drawing a gene annotated for a particular function from the entire genome of interest without even considering the clustering results based on differential DNA expression levels.

### Computational resources

All optimization formulations are written in GAMS (General Algebraic Modeling System) [54] and solved using the commercial solver CPLEX 8.0. GAMS is a high level modeling system specifically designed for mathematical optimization. It consists of a language compiler and an integrated high performance solver such as CPLEX, DICOPT, or XPRESS.

## Authors' contributions

MPT conceived of the study, implemented the computational coding and quantitative analysis, and drafted the manuscript. ENS performed the FIRE analysis and linkage studies of datasets II and III, and refined the manuscript. JRB carried out the microarray experiments for datasets I and II, and refined the manuscript. CAF conceived of the study, analyzed the quantitative results, and refined the manuscript. All authors read and approved the final manuscript.

## Supplementary Material

Additional file 1 Figure legends for three supplementary figures Click here for file

Additional file 2 Sample plots from iterative clustering of dataset II Click here for file

Additional file 3 Percentile of genes in dataset II below a particular fold change Click here for file

Additional file 4 Percentile of genes in dataset II below a particular fold change Click here for file

Additional file 5 Availability of datasets I, II and III Click here for file

## References

[B1] Allison DB, Cui X, Page GP, Sabripour M (2006). Microarray data analysis: from disarray to consolidation and consensus. Nat Rev Genet.

[B2] Gasch AP, Spellman PT, Kao CM, Carmel-Harel O, Eisen MB, Storz G, Botstein D, Brown PO (2000). Genomic expression programs in the response of yeast cells to environmental changes. Mol Biol Cell.

[B3] Lin X, Floudas CA, Wang Y, Broach JR (2003). Theoretical and computational studies of the glucose signaling pathways in yeast using global gene expression data. Biotechnol Bioeng.

[B4] Sokal RR, Michener CD (1958). A statistical method for evaluating systematic relationships. Univ Kans Sci Bull.

[B5] Hartigan JA, Wong MA (1979). Algorithm AS 136: A K-means clustering algorithm. Appl Stat J Roy St C.

[B6] Zhang B, Hsu M, Dayal U (1999). K-harmonic means – A data clustering algorithm. Hewlett Packard Research Laboratory Technical Report.

[B7] Likas A, Vlassis N, Vebeek JL (2003). The global K-means clustering algorithm. Pattern Recogn.

[B8] Adams WP, Sherali HD (1990). Linearization strategies for a class of zero-one mixed integer programming problems. Operations Research.

[B9] Sherali HD (2005). A global optimization RLT-based approach for solving the hard clustering problem. Journal of Global Optimization.

[B10] Tan MP, Broach JR, Floudas CA (2007). A novel clustering approach and prediction of optimum number of clusters: Global optimum search with enhanced positioning. Journal of Global Optimization.

[B11] Ruspini EH (1969). A new approach to clustering. Inf Control.

[B12] Sherali HD, Desai J (2005). A global optimization RLT-based approach for solving the fuzzy clustering problem. Journal of Global Optimization.

[B13] Heyer LJ, Kruglyak S, Yooseph S (1999). Exploring expression data: identification and analysis of co-expressed genes. Genome Res.

[B14] Kohonen T (1997). Self-Organizing Maps.

[B15] Herrero J, Valencia A, Dopazo J (2001). A hierarchical unsupervised growing neural network for clustering gene expression patterns. Bioinformatics.

[B16] Tishby N, Pereira F, Bialek W (1999). The information bottleneck method. Proceedings of the 37th Annual Allerton Conference on Communication, Control Comput.

[B17] Slonim N, Atwal GS, Tkacik G, Bialek W (2005). Information-based clustering. Proc Natl Acad Sci USA.

[B18] Kirkpatrick S, Gelatt CD, Vecchi MP (1983). Optimization by simulated annealing. Science.

[B19] Lukashin AV, Fuchs R (2001). Analysis of temporal gene expression profiles: clustering by simulated annealing and determining the optimal number of clusters. Bioinformatics.

[B20] Jiang D, Tang C, Zhang A (2004). Cluster analysis for gene expression data: A survey. IEEE Transactions on Knowledge and Data Engineering.

[B21] Yeung KY, Haynor DR, Ruzzo WL (2001). Validating clustering for gene expression data. Bioinformatics.

[B22] Davies DL, Bouldin DW (1979). A cluster separation measure. IEEE Trans Pattern Anal Machine Intell.

[B23] Pavlidis P, Qin J, Arango V, Mann JJ, Sibille E (2004). Using the gene ontology for microarray data mining: a comparison of methods and application to age effects in human prefrontal cortex. Neurochem Res.

[B24] Guldener U, Munsterkotter M, Kastenmuller G, Strack N, van Helden J, Lemer C, Richelles J, Wodak SJ, Garcia-Martinez J, Perez-Ortin JE (2005). CYGD: the Comprehensive Yeast Genome Database. Nucleic Acids Res.

[B25] The Gene Ontology Consortium (2000). Gene ontology: tool for unification of biology. Nat Genet.

[B26] Cheng J, Cline M, Martin J, Finkelstein D, Awad T, Kulp D, Siani-Rose MA (2004). A knowledge-based clustering algorithm driven by gene ontology. J Biopharm Stat.

[B27] Pan W (2006). Incorporating gene functions as priors in model-based clustering of microarray gene expression data. Bioinformatics.

[B28] Komura D, Nakamura H, Tsutsumi S, Aburatani H, Ihara S (2004). Incorporating prior knowledge into clustering of gene expression profiles. 15th International Conference on Genome Informatics.

[B29] Dotan-Cohen D, Melkman AA, Kasif S (2007). Hierarchical tree snipping: clustering guided by prior knowledge. Bioinformatics.

[B30] Huang D, Pan W (2006). Incorporating biological knowledge into distance-based clustering analysis of microarray gene expression data. Bioinformatics.

[B31] Floudas CA, Aggarwal A, Ciric AR (1989). Global optimum search for non convex NLP and MINLP problems. Comp Chem Eng.

[B32] Paules GE, Floudas CA (1989). APROS – Algorithmic development for discrete-continuous optimization problems. Operations Research.

[B33] Floudas CA (1995). Nonlinear and Mixed-Integer Optimization: Fundamentals and Applications.

[B34] Wang Y, Pierce M, Schneper L, Guldal CG, Zhang X, Tavazoie S, Broach JR (2004). Ras and Gpa2 mediate one branch of a redundant glucose signaling pathway in yeast. PLoS Biol.

[B35] Broach JR, Deschenes RJ (1990). The function of ras genes in Saccharomyces cerevisiae. Adv Cancer Res.

[B36] Schneper L, Duvel K, Broach JR (2004). Sense and sensibility: nutritional response and signal integration in yeast. Curr Opin Microbiol.

[B37] Santangelo GM (2006). Glucose signaling in Saccharomyces cerevisiae. Microbiol Mol Biol Rev.

[B38] Carlson M (1999). Glucose repression in yeast. Curr Opin Microbiol.

[B39] Johnston M, Kim JH (2005). Glucose as a hormone: receptor-mediated glucose sensing in the yeast Saccharomyces cerevisiae. Biochem Soc Trans.

[B40] Crauwels M, Donaton MC, Pernambuco MB, Winderickx J, de Winde JH, Thevelein JM (1997). The Sch9 protein kinase in the yeast Saccharomyces cerevisiae controls cAPK activity and is required for nitrogen activation of the fermentable-growth-medium-induced (FGM) pathway. Microbiology.

[B41] Elemento O, Slonim N, Tavazoie S Uncovering regulatory elements from expression data using mutual information. Mol Cell.

[B42] Myers CL, Barrett DR, Hibbs MA, Huttenhower C, Troyanskaya OG (2006). Finding function: evaluation methods for functional genomic data. BMC Genomics.

[B43] Brem RB, Yvert G, Clinton R, Kruglyak L (2002). Genetic dissection of transcriptional regulation in budding yeast. Science.

[B44] Yvert G, Brem RB, Whittle J, Akey JM, Foss E, Smith EN, Mackelprang R, Kruglyak L (2003). Trans-acting regulatory variation in Saccharomyces cerevisiae and the role of transcription factors. Nat Genet.

[B45] Foat BC, Houshmandi SS, Olivas WM, Bussemaker HJ (2005). Profiling condition-specific, genome-wide regulation of mRNA stability in yeast. Proc Natl Acad Sci USA.

[B46] The Princeton University Microarray Database. http://puma.princeton.edu.

[B47] Gollub J, Ball Ca, Binkley G, Demeter J, Finkelstein DB, Hebert JM, Hernandez-Boussard T, Jin H, Kaloper M, Matese JC, Schroeder M, Brown PO, Botstein D, Sherlock G (2003). The Stanford microarray database: data access and quality assessment tools. Nuclei Acids Res.

[B48] Troyanskaya O, Cantor M, Sherlock G, Brown P, Hastie T, Tibshirani R, Botstein S, Altman RB (2001). Missing value estimation methods for DNA microarrays. Bioinformatics.

[B49] Tan MP, Broach JR, Floudas CA (2007). Evaluation of normalization and pre-clustering issues in a novel clustering approach: global optimum search with enhanced positioning. J Bioinform Comput Biol.

[B50] The Saccharomyces Genome Database. http://www.yeastgenome.org.

[B51] Broman KW, Wu H, Sen S, Churchill GA (2003). R/qtl: QTL mapping in experimental crosses. Bioinformatics.

[B52] Churchill GA, Doerge RW (1994). Empirical threshold values for quantitative trait mapping. Genetics.

[B53] http://function.princeton.edu/GRIFn.

[B54] Brooke A, Kendrick D, Meeraus A (1988). GAMS: A User's Guide.

