# Supplementary material for: Microarray data mining: A novel optimization-based approach to uncover biologically coherent structures
Source: BMC Bioinformatics. 2008 Jun 6;9:268. doi: 10.1186/1471-2105-9-268 (PMC2442101; doi:10.1186/1471-2105-9-268)
Supplement: Additional file 1 — Figure legends for three supplementary figures [file 1471-2105-9-268-S1.pdf]

Supplementary material – figure legends

(Additional File 1)

Figure S1. Sample plots from iterative clustering of dataset II

(See Additional File 2)

We show three sample gene expression time course plots out of the 38 high-quality clusters formed from iteratively refining the clustered results of dataset II. We present clusters of varying sizes to demonstrate that the iterative approach does not contain size-bias.

Figure S2. Percentile of genes in dataset II below a particular fold change

(See Additional File 3)

Each colored line represents a particular time point out of the 76 features for dataset II. The plot shows the cumulative percentage of the 5657 genes that are below a particular fold change log ratio. The darkened line is the mean regression line, and shows that the requirement that every gene shows a ‘significant’ level of differential mRNA expression for at least 10% of the time points (or roughly over two experiments) translates to an average fold change of about 1.7.

Figure S3. Percentile of genes in dataset II below a particular fold change (See Additional File 4)

For each fold change ratio line, the number of genes in dataset II with a minimum percentage of time points meeting the fold change criterion is shown. The feasible region is formed by (I) considering a two standard deviation interval around the number of genes that show average expression intensities of over 100, a level recommended by the Agilent DNA microarray for expression significance, and (II) taking a 1.5-2 fold-change requirement for the genes, which translates to a minimum of 5-20% of all time points. The darkened dots represent the sets that will be considered for parametric testing to find the most suitable fold-change criteria for clustering dataset II using the EP_GOS_Clust.
