# Supplementary figures and images for: Microarray data mining: A novel optimization-based approach to uncover biologically coherent structures
Source: BMC Bioinformatics. 2008 Jun 6;9:268. doi: 10.1186/1471-2105-9-268 (PMC2442101; doi:10.1186/1471-2105-9-268)

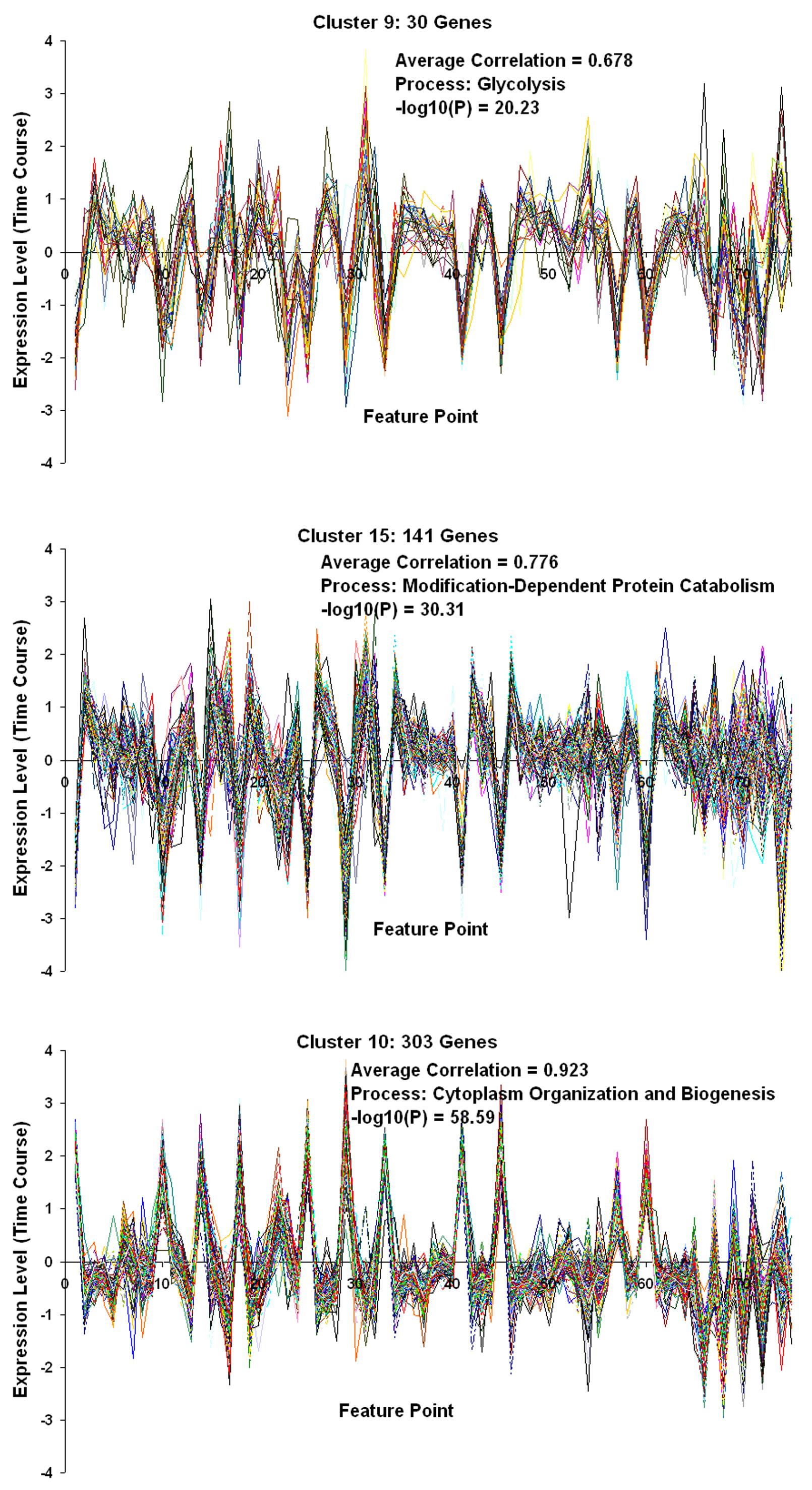

Supplement: Additional file 2 — Sample plots from iterative clustering of dataset II [file 1471-2105-9-268-S2.jpeg]

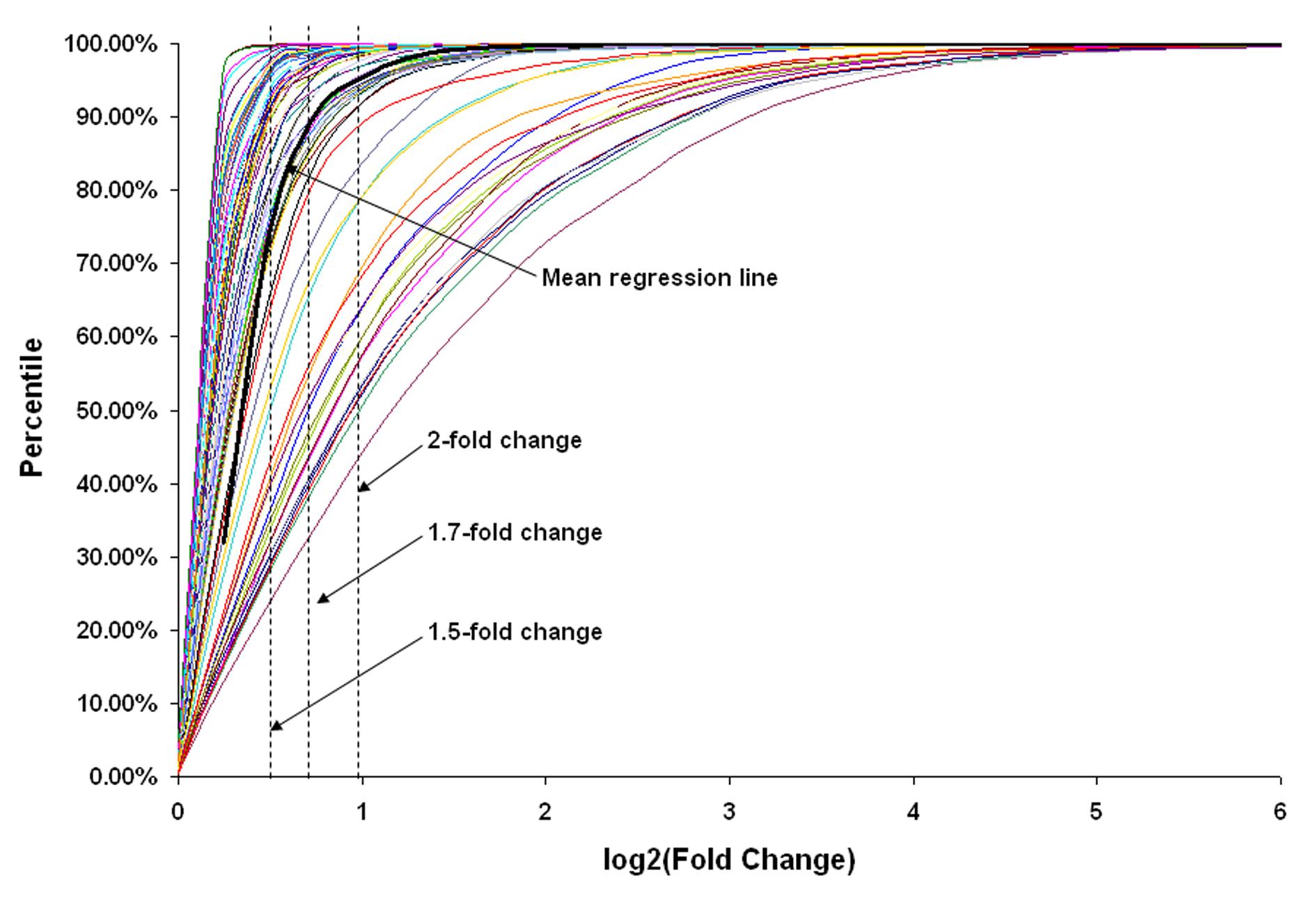

Supplement: Additional file 3 — Percentile of genes in dataset II below a particular fold change [file 1471-2105-9-268-S3.jpeg]

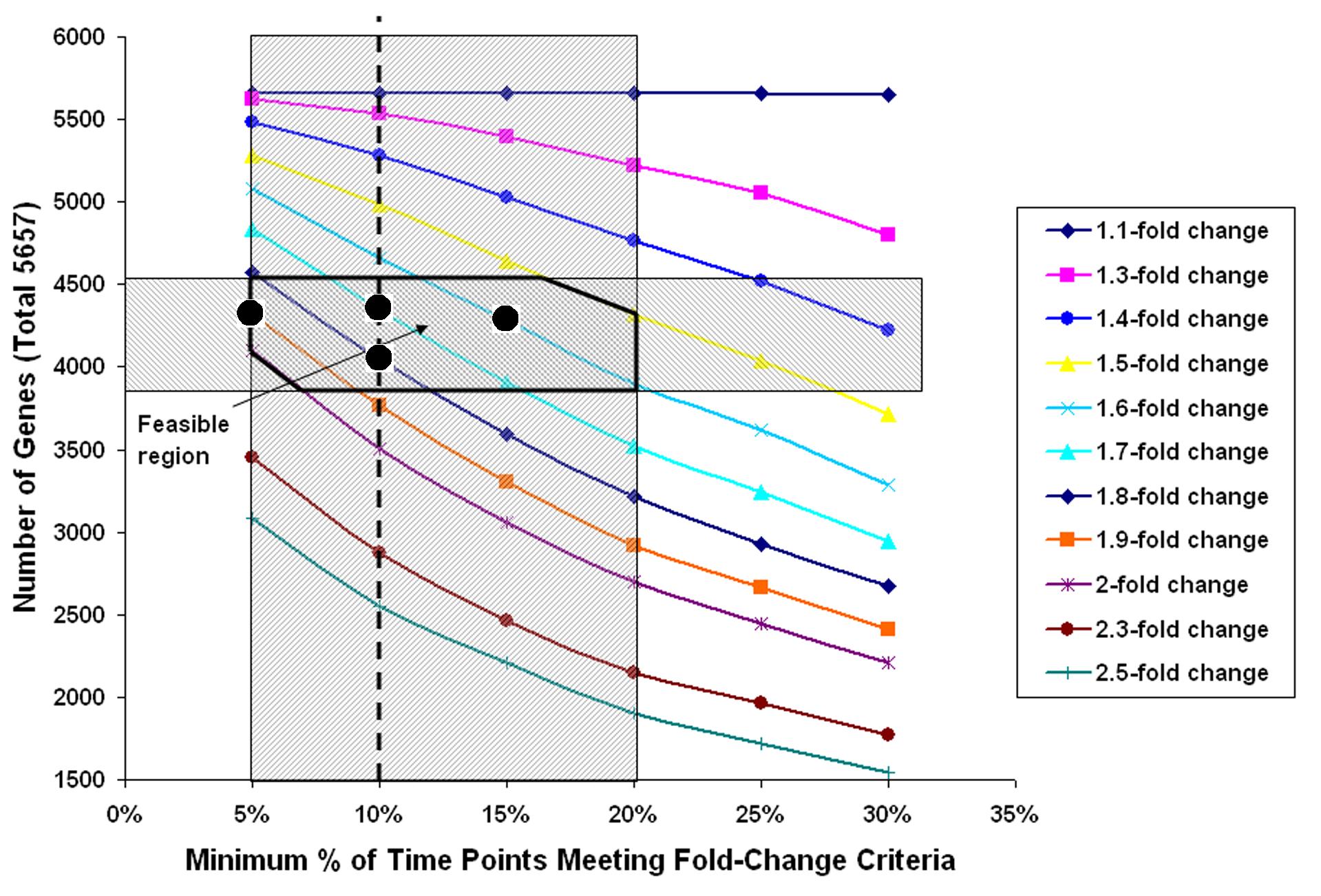

Supplement: Additional file 4 — Percentile of genes in dataset II below a particular fold change [file 1471-2105-9-268-S4.jpeg]
