# Supplementary material for: Microarray data mining: A novel optimization-based approach to uncover biologically coherent structures
Source: BMC Bioinformatics. 2008 Jun 6;9:268. doi: 10.1186/1471-2105-9-268 (PMC2442101; doi:10.1186/1471-2105-9-268)
Supplement: Additional file 5 — Availability of datasets I, II and III [file 1471-2105-9-268-S5.pdf]

Additional file 5 – Availability of datasets

Dataset I

Available for download from [http://biology.plosjournals.org/archive/1545-7885/2/5/supinfo/10.1371_journal.pbio.0020128.st001.txt]. The citation is Wang et al., PLoS Biology 2(5):E128 (2004).

Dataset II

Available publicly from PUMAdb, the Princeton University microarray database [http://puma.princeton.edu]. The link is [http://puma.princeton.edu/cgi-bin/exptsets/viewExptSets.pl?exptset_no=5078].

Dataset III

Reported in Brem and Kruglyak, PNAS 102:1572 (2005) and is deposited in the Gene Expression Omnibus database (accession no. GSE1990 [NCBI GEO]).
